# Cheerfulness

**Published:** 1866-07

**Authors:** 


### CHEERFULNESS.

A hearty laugh is known the world over to be a health promoter ; it elevates the spirits, enlivens the circulation, and is marvelously contagious in a good sense. A poor, miserable and vulgar-minded croaker may by a single ill-tempered remark cloud a whole company in a drawing-room or at the table, while a cheery minded jovial nature would diffuse sunshine and geniality by the utterance of a timely jest, a ludicrous sentiment or a preposterous bon mot. Travellers "snowed-up" on a rail track, "becalmed at sea," "brought to" on sand bar, have many a time witnessed an instantaneous chang from sombre impatience and anxiety to uncontrollable mirt in which the most desperate "blue" was forced to join, l some happy hit of an irrepressible fellow passenger. It would be well for every one to lay in a store of "good things," and keep that store well replenished, to draw therefrom things new and old in the way of jokes, ludicrous, laughable and instructive. If all would do this it would nullify many of the disagreeables of travelling and make "detentions" delightful.

